# The Role of Matrix Metalloproteinases (MMP-2 and MMP-9) in Ageing and Longevity: Focus on Sicilian Long-Living Individuals (LLIs)

**DOI:** 10.1155/2020/8635158

**Published:** 2020-05-05

**Authors:** Patrizia Cancemi, Anna Aiello, Giulia Accardi, Rosalia Caldarella, Giuseppina Candore, Calogero Caruso, Marcello Ciaccio, Laura Cristaldi, Francesca Di Gaudio, Valentina Siino, Sonya Vasto

**Affiliations:** ^1^Department of Biological Chemical and Pharmaceutical Sciences and Technologies, University of Palermo, Palermo, Italy; ^2^Laboratory of Immunopathology and Immunosenescence, Department of Biomedicine, Neurosciences and Advanced Diagnostics, University of Palermo, Palermo, Italy; ^3^Department of Laboratory Medicine, “P. Giaccone” Palermo University Hospital, Palermo, Italy; ^4^Unit of Clinical Biochemistry, Clinical Molecular Medicine and Laboratory Medicine, Department of Biomedicine, Neurosciences and Advanced Diagnostics, University of Palermo, Palermo, Italy; ^5^Institute for Biomedical Research and Innovation, National Research Council of Italy, Palermo, Italy; ^6^Department of Health Promotion, Mother and Child Care, Internal Medicine and Medical Specialties, University of Palermo, Palermo, Italy; ^7^Department of Immunotechnology, Lund University, Medicon Village, Lund, Sweden

## Abstract

Extracellular matrix metalloproteinases (MMPs) are a group of proteins that activate substrates by enzymatic cleavage and, on the basis of their activities, have been demonstrated to play a role in ageing. Thus, in order to gain insight into the pathophysiology of ageing and to identify new markers of longevity, we analysed the activity levels of MMP-2 and MMP-9 in association with some relevant haematochemical parameters in a Sicilian population, including long-living individuals (LLIs, ≥95 years old). A cohort of 154 healthy subjects (72 men and 82 women) of different ages (age range 20-112) was recruited. The cohort was divided into five subgroups: the first group with subjects less than 40 years old, the second group ranging from 40 to 64 years old, the third group ranging from 65 to 89 years old, the fourth group ranging from 90 to 94 years old, and the fifth group with subjects more than 95 years old. A relationship was observed between LLIs and MMP-2, but not between LLIs and MMP-9. However, in the LLI group, MMP-2 and MMP-9 values were significantly correlated. Furthermore, in LLIs, we found a positive correlation of MMP-2 with the antioxidant catabolite uric acid and a negative correlation with the inflammatory marker C-reactive protein. Finally, in LLIs MMP-9 values correlated directly both with cholesterol and with low-density lipoproteins. On the whole, our data suggest that the observed increase of MMP-2 in LLIs might play a positive role in the attainment of longevity. This is the first study that shows that serum activity of MMP-2 is increased in LLIs as compared to younger subjects. As far as we are concerned, it is difficult to make wide-ranging conclusions/assumptions based on these observations in view of the relatively small sample size of LLIs. However, this is an important starting point. Larger-scale future studies will be required to clarify these findings including the link with other systemic inflammatory and antioxidant markers.

## 1. Introduction

Ageing is a time-dependent functional decline, which involves a progressive deterioration of organism physiological functions heading to increased susceptibility to disease and death. This process is unavoidable and extremely complex. However, in the last 40 years, a lot of efforts have been made to characterize ageing. As described in literature, there are two ways to age. The first is free of age-related diseases and without disability (successful ageing), while the latter is characterized by a progressive tendency toward inflammageing, disability, and age-related diseases (unsuccessful ageing) [1]. Major age-related diseases include atherosclerosis, Alzheimer's disease, and diabetes, where the inflammatory components that were prolonged and persisted become damaging [1, 2]. On the other hand, long-living individuals (LLIs) are considered the best example of successful ageing [3].

Extracellular proteinases are a group of proteins that activate substrates by enzymatic cleavage and, on the basis of working mechanisms, are classified into aspartic, metallo-, cysteine, serine, and threonine proteinases [4]. Among different immune cells, macrophage and neutrophils are the main responsible for matrix metalloproteinase (MMP) production. This group of proteins controls a large variety of key physiological and pathological processes, including tissue remodelling, DNA replication, cell-cycle progression, neurodegeneration, and cancer [5].

Moreover, MMPs are responsible for remodelling of extracellular matrix (ECM), which represents a three-dimensional network of extracellular macromolecules such as collagen, enzymes, and glycoproteins, that provides structural and biochemical support of surrounding cells [6], particularly of stem cell niche [7, 8]. The perturbation of ECM remodelling has been associated with ageing and age-related disorders, for example, progeria (an extremely rare, autosomal, dominant genetic disorder in which symptoms, resembling aspects of ageing, are manifested at a very early age) [9], arterial ageing, hypertension-associated vascular changes [10], cancer metastasis, heart failure, and cerebral ischemia and neurological disorders including Parkinson's and Alzheimer's diseases [11, 12]. Therefore, the analysis of MMPs can add important information to ageing process and to longevity. As an example, ECM proteins, like elastins, can be a good candidate as a biomarker of ageing due to low turnover and to capacity to accumulate damage during ageing process [13], as in a vascular one. Also, collagen, another ECM protein, is deeply involved in ageing since its accumulation drives both vascular and lung ageing [14]. In addition, in *Caenorhabditis elegans*, collagen, among other ECM proteins, is influenced by the insulin/IGF-1-like signalling, which in turn extend life span in a worm model [15].

MMP-2 (a type of gelatinase A, 72 kDa) and MMP-9 (a type of gelatinase B, 92 kDa) are composed of 3 domains, distinguished by the presence of type II additional fibronectin domain inserted into the catalytic domain. They are able to degrade collagen, elastin, fibronectin, gelatin, and laminin and have both proinflammatory and anti-inflammatory impacts on numerous tissues [16–18]. In particular, MMP-2 is constitutively expressed in several tissues and is regulated by tumor necrosis factor-*α* under the influence of NF-*κ*B transcription factor [19], while redox-regulated p38 phosphorylation and subsequent AP-1 activation appear to be critical for lipopolysaccharide-induced MMP-9 expression, at least in murine macrophages [20]. MMP-2 is tightly associated with inflammatory states such as osteoarthritis [21, 22]; besides, MMP-2 protects from hypertensive heart disease by suppressing the transcription and activity of 3-hydroxy-3-methylglutaryl-CoA reductase in the early stages of the hypertensive response [23]. Interestingly, it was demonstrated that in atherosclerotic plaque, MMP-2 levels decreased, compared to nonatherosclerotic human tissues [24], whereas MMP-9 levels increased, showing that MMP-9 activity contributes to endothelial dysfunction [25].

MMP-9 is also implicated in lipid metabolism [26], and in a mouse model, it plays an important role for aneurysm formation [27, 28]. Moreover, the protein levels are detected in the acute phase after stroke, whereas MMP-2 protein levels were increased several days after when barrier leakage is presumably restored.

Thus, in our study, in order to gain insight into ageing, age-related disease, and longevity, the activity levels of MMP-2 and MMP-9 were analysed in association with some relevant haematochemical parameters in a Sicilian population.

## 2. Materials and Methods

### 2.1. Subject Recruitment and Study Design

A cohort of 154 healthy subjects (72 men and 82 women) of different ages (age range 20-112) was recruited. Donors were all Sicilians, living in Western Sicily. A group of well-trained nutritionists and physicians administered a questionnaire to collect demographic and anamnestic data of interest. Participants were selected on the basis of their health status since none of them had neoplastic, infective, or autoimmune diseases and none was prescribed drugs known to interfere with immune-inflammatory responses. Participants (or their relatives for some LLIs) signed an informed consent before the enrolment. To respect the privacy, everyone was identified with an alphanumeric code. A database was created to handle the collected information. The study protocol, conducted in accordance with the Declaration of Helsinki and its amendments, was approved by the Ethics Committee of Palermo University Hospital (Nutrition and Longevity, No. 032017). The suitability of the sample size was checked using free software (http://ps-powerand-sample-sizecalculation.software.informer.com) on the basis of the results of our previous studies. The analysed cohort was divided into five subgroups: the first group included people less than 40 years old (19%, group 1—young people; *N* = 29), the second group ranging from 40 to 64 years old (25%, group 2—adult people; *N* = 39), the third group ranging from 65 to 89 years old (32%, group 3—old people; *N* = 50), the fourth group ranging from 90 to 94 years old (8%, group 4—oldest old people; *N* = 12), and the fifth group characterized by subjects 95 or more years old (16%, group 5—formed by LLIs; *N* = 24). The recruitment was performed in accordance with the relevant guidelines and regulations.

The recruited participants underwent vein puncture after a fasting period of 10–12 hours. The fasting blood samples were obtained in the morning (between 8:30 and 10 a.m.) and were collected in serum tubes with no additives.

The following haematochemical parameters were performed for all participants: albumin, alanine and aspartate transaminases, alkaline phosphatase, bilirubin, calcium, creatinine, C-reactive protein (CRP), ferritin, glycaemia, high-density lipoproteins, iron, low-density lipoproteins (LDL), magnesium, potassium, total cholesterol (CHO), total proteins, transferrin, triglycerides, urea, and uric acid (UA) as well as complete blood count tests. The tests were carried out at the Department of Laboratory Medicine, “P. Giaccone” University Hospital, Palermo, according to standard procedures. In particular, CRP measurement was performed by immunoturbidimetry methods, UA by colorimetric test, and lipid parameters by enzymatic colorimetric test through Roche/Hitachi Cobas system.

Table 1 shows the baseline characteristics of the studied cohort with haematochemical parameters shown to be associated with MMPs.

### 2.2. Gelatin Zymography and Polyacrylamide Gel Electrophoresis

**Figure 1:**
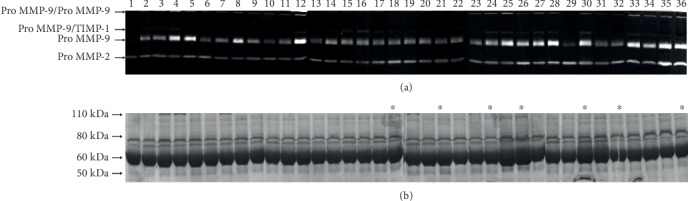
Pro-MMP-2 and Pro-MMP-9 activity levels evaluated by gelatin zymography. (a) Prototype of gelatin zymography of 36 randomly selected serum samples used. (b) SDS-PAGE electrophoresis of the same serum samples of (a) stained with Coomassie Blue. LLIs are indicated in (b) with asterisks.

**Figure 2:**
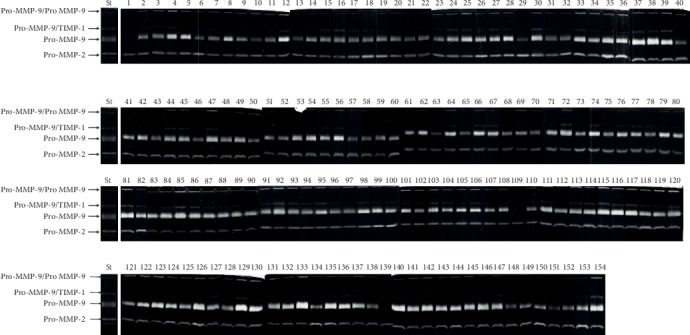
Gelatin zymography of the 154 analysed serum samples. Each lane represents a different subject. Experiments were performed in triplicate, and the densitometric bands were quantified by using ImageJ software. St represents protein samples extracted from human breast cancer tissues used as standard.

Serum protein concentration was quantified spectrophotometrically, using the Bradford assay, as described in [29, 30]. For SDS-PAGE, aliquots of 10 *μ*L of sera, previously diluted (1 : 25), containing approximately 28 *μg* of total proteins, were mixed with Laemmli buffer (2% *w*/*v* SDS, 10% glycerol, 5% *β*-mercaptoethanol, and 62 mM Tris-HCl pH 6.8), boiled for 5 min, and loaded on 10 × 8 cm vertical 8% polyacrylamide gel. The run was performed at 150 V for 50 min with a Mini Protean II Xi System (Bio-Rad Laboratories S.r.l., Milano). The running buffer was 25 mM Tris-HCl, 200 mM glycine, and 0.1% *w*/*v* SDS. Gels were stained with 0.2% Coomassie Brilliant Blue G-250 in 40% methanol and 10% acetic acid and de-stained in 7% methanol and 5% acetic acid. For zymography assay, aliquots of 10 *μ*L of sera previously diluted (1 : 25) were loaded onto 7.5% polyacrylamide SDS-PAGE gel copolymerized with 0.1% gelatin, under nonreducing conditions, and run at 150 V in a Tris-glycine buffer, as described by Cancemi et al. [31–33]. After electrophoresis, gels were incubated at room temperature for 1 h in a wash buffer (50 mM Tris-HCl pH 7.5 and 2.5% Triton X-100) to remove the SDS and then incubated for 18 h at 37°C with an activation buffer (50 mM Tris-HCl pH 7.5, 150 mM NaCl, and 5 mM CaCl_2_), to allow the activation of the gelatinases, as previously described [34, 35]. Gels were stained with Coomassie Blue. Band intensity was measured with ImageJ software. Protein samples extracted from human breast cancer tissues [36–39] were used as reference for MMP-2 and MMP-9 activity levels. All experiments were performed in triplicate. Gelatinolytic activity appeared as white bands on a dark background (Figures 1(a) and 2).

### 2.3. Statistical Analysis

The data were processed using GraphPad Prism software version 5.0 (GraphPad Software, La Jolla, CA, United States). Nonparametric tests were applied for statistical analyses. In particular, the Mann–Whitney *U* test was used to compare two subgroups of patients, and the Kruskal–Wallis test was used to compare three or more subgroups of subjects. The two-tailed alpha level was set to *p* < 0.05 to indicate a significant difference. The correlations were performed applying the Pearson correlation test.

## 3. Results

### 3.1. Activity Levels of MMP-2 and MMP-9 in Serum Samples and Their Correlation with Age

Sera from all subjects enrolled in this study were subjected to gelatin zymography to determine the relative levels of activity attributable to MMP-2 and MMP-9.  Figure 1 shows a panel of 36 zymograms randomly selected among the collection of our samples. Parallel SDS polyacrylamide gels were run in order to ascertain the correct protein loading. The two prominent gelatinolytic bands represent the proenzymatic forms of MMP-2 (Pro-MMP-2) (72 kDa) and of MMP-9 (Pro-MMP-9) (92 kDa). Two additional lytic bands of 200 and 116 kDa, identified as MMP-9 dimers (220 kDa) and as MMP-9/TIMP1 complex (116 kDa), are also evident [40, 41]. In all serum samples, no activity levels were evident for the active forms of the MMPs.

The activity levels of Pro-MMP-9 in all samples appear more intense than Pro-MMP-2. In order to assess the relative variations of the two Pro-MMP levels in all subjects (Figure 2), gels, containing samples, run in triplicate were subjected to densitometric analysis by using the ImageJ software, as described in Materials and Methods.

The subjects are allocated in the gels as follows: group 1—young people, *N* = 29: lanes 12, 44, 45, 46, 75, 77, 79, 80, 81, 82, 85, 89, 90, 92, 98, 99, 100, 106, 109, 110, 115, 118, 122, 125, 128, 130, 136, 142, and 145; group 2—adult people, *N* = 39: lanes 11, 14, 15, 16, 25, 27, 33, 34, 39, 50, 52, 54, 60, 64, 66, 69, 72, 74, 78, 88, 91, 93, 95, 104, 105, 108, 116, 117, 121, 123, 124, 127, 131, 134, 135, 138, 139, 144, and 152; group 3—old people, *N* = 50: lanes 1, 2, 3, 4, 5, 6, 7, 8, 9, 10, 13, 17, 19, 20, 22, 23, 28, 29, 38, 42, 43, 47, 51, 55, 57, 63, 68, 76, 83, 84, 86, 87, 94, 97, 101, 102, 103, 113, 114, 119, 120, 126, 132, 137, 140, 141, 146, 149, 151, and 154; group 4—oldest old people, *N* = 12: lanes 31, 35, 53, 56, 58, 61, 62, 70, 71, 73, 129, 133; and group 5—LLIs, *N* = 24: lanes 18, 21, 24, 26, 30, 32, 36, 37, 40, 41, 48, 49, 59, 65, 67, 96, 107, 111, 112, 143, 147, 148, 150, and 153.

**Figure 3:**
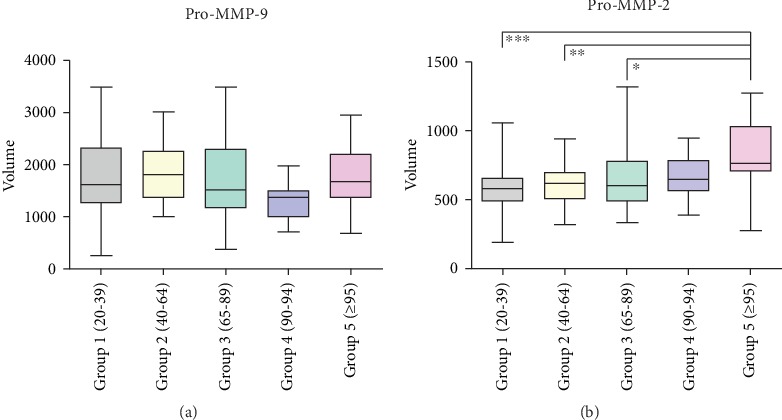
Box plot graphs of Pro-MMP-9 (a) and Pro-MMP-2 (b) activity levels evaluated by gelatin zymography grouped for age. The ends of the box are the upper and lower quartiles, the lines extending parallel from the boxes are used to indicate variability outside the upper and lower quartiles while the median is marked by a vertical line inside the box. The densitometric analysis, performed by measuring the intensity levels of each band and the corresponding area, is referred as volume. Statistical analysis was performed applying the Kruskal–Wallis nonparametric test. ^∗^*p* < 0.05, ^∗∗^*p* < 0.01, and ^∗∗∗^*p* < 0.001.

In order to assess the possible correlations between levels of gelatinase activity and age, a statistical analysis was performed between the enzymatic activities of the different individuals divided in groups as described in Materials and Methods. Figures 3(a) and 3(b) report an overview of the distribution of Pro-MMP-2 and Pro-MMP-9 activities over the 5 groups, corresponding to the average of three measurements per sample. Although the activity levels of each Pro-MMP are variable within the subjects, a significant association was obtained for Pro-MMP-2 (Figure 3(b)). In particular, the Pro-MMP-2 activity levels increase with age. Significant differences were observed between the first, second, and third groups and the group of LLIs. No significant differences were obtained between the fourth and the fifth group, probably due to poor representation of the fourth group (8%) and the age proximity with the fifth group.

In contrast, the activity levels of Pro-MMP-9 (Figure 3(a)) did not show any correlation with age. However, only in the LLI group the activity level of Pro-MMP-2 was significantly related to Pro-MMP-9 activity (*r* = 0.53, *p* < 0.01).

**Figure 4:**
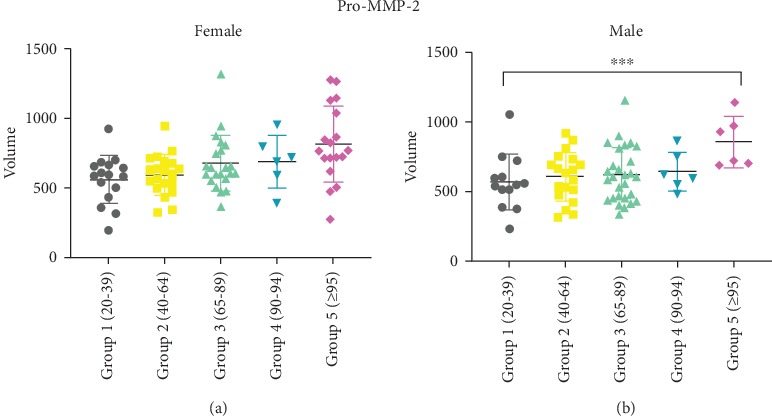
Scatter plot graphs of Pro-MMP-2 activity levels in female (a) and male (b) evaluated by gelatin zymography and grouped for age. The lines indicate mean and standard error. The densitometric analysis, performed by measuring the intensity levels of each band and the corresponding area, is referred as volume. Statistical analysis was performed applying the Kruskal–Wallis nonparametric test. ^∗∗∗^*p* < 0.001.

Interestingly, when the cohorts were analysed according to gender, a clear increase of Pro-MMP-2 activity with age was observed in the male gender but not in female gender (Figures 4(a) and 4(b)), probably because, especially in women, the expression of MMPs is also dependent on the hormonal status [42, 43]. Another hypothesis could be that of the better health of male LLIs. It is known that male centenarians are fewer in number but healthier than women [44]. The small number studied does not allow us to analyse this possibility. Due to the reduced size samples, in males, the significance was only obtained between the LLI and younger groups.

### 3.2. Pro-MMP-2 and Pro-MMP-9 Correlation with Some Relevant Haematochemical Parameters

**Figure 5:**
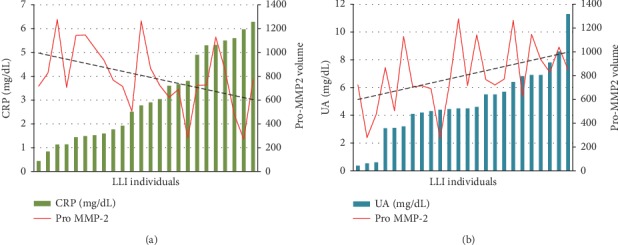
Histograms of Pro-MMP-2 activity levels in the LLI group and hematological values of CRP (a) and UA (b) to show the found correlations. The trend of the line in hematological parameters shows a positive or inverse correlation.

The correlation between the serum activity levels of Pro-MMP-2 and CRP and UA was also analysed. In fact, MMP-2 activity plays a relevant role in tissue remodelling and in the pathophysiology of inflammation, CRP is an inflammatory marker [45], and serum UA is considered to have an antioxidant effect [46]. Interestingly, a significant correlation was found only in LLIs. In particular, we found an inverse correlation between CRP levels and Pro-MMP-2 activity in LLIs (*r* = −0.39, *p* < 0.05; Figure 5(a)). On the contrary, we found a positive correlation between UA levels and Pro-MMP-2 activity in LLIs (*r* = 0.41, *p* < 0.05; Figure 5(b)).

**Figure 6:**
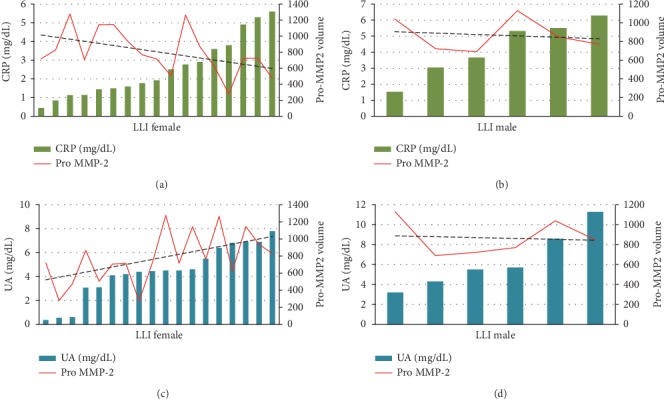
Histograms of Pro-MMP-2 activity levels in the LLI group and hematological values of CRP (a, b) and UA (c, d) in female (a–c) and in male (b–d) to show the found correlations. The trend of the line in hematological parameters shows a positive or inverse correlation.

The data in Figure 6 are depicted also according to the gender where it is clear that in the female LLI population, Pro-MMP-2 activity inversely correlates with CRP (*r* = −0.5680, *p* = 0.0139) and positively correlates with UA (*p* = 0.512, *p* = 0.0305).

Since MMP-9 modulates cholesterol metabolism [26], we analysed the correlation between Pro-MMP-9 activity and CHO levels. Interestingly, we found a positive correlation between cholesterol levels and Pro-MMP-9 activity in LLIs (*r* = 0.52, *p* < 0.001).

**Figure 7:**
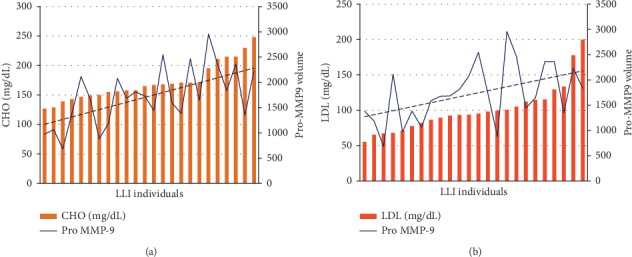
Histograms of Pro-MMP-9 (a, b) activity levels in the LLI group and hematological values of CHO (a) and LDL (b) to show the found correlations. The trend of the line in hematological parameters shows a positive correlation for both.

We also found a positive correlation between LDL levels and Pro-MMP-9 activity in LLIs (*r* = 0.43, *p* < 0.05; Figures 7(a) and 7(b)).

**Figure 8:**
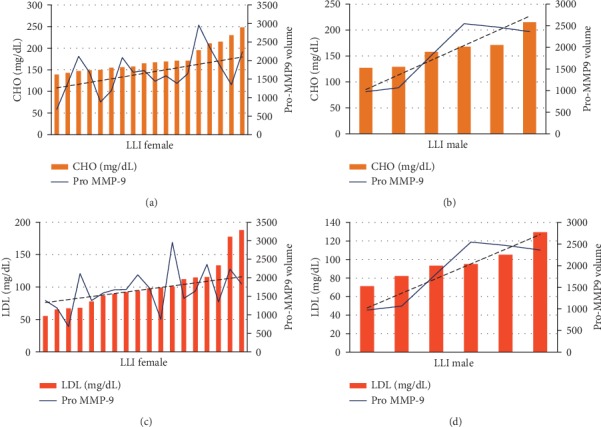
Histograms of Pro-MMP-9 activity levels and hematological values of CHO in the female (a) and male (b) LLI group and of LDL in the female (c) and male (d) LLI group.

The data in Figure 8 show the same results according to gender. In the male LLI population, Pro-MMP-9 activity correlates positively with CHO (*r* = 0.81, *p* = 0.0479) and with LDL (*p* = 0.77, *p* = 0.049).

## 4. Discussion

In the present study, the possible role played by MMPs in ageing and longevity has been focused. MMPs are a family of structurally and functionally related zinc-dependent proteases with a wide range of substrates, including ECM components, cytokines, receptors, and cell motility factors. It is widely recognized that MMPs play a role in the pathophysiology of various tissues during growth, development, and ageing. Traditionally, the gelatinase members of the MMP family, MMP-2 and MMP-9, have been the easiest to detect using gelatin zymography; therefore, there are much more available data on them [47–49].

Previous studies demonstrated that ageing is associated with increased activities of MMP-2 [50] or of MMP-2 and MMP-9 [51] and concentration of active MMP-9 decreases with age [48].

Many studies on ageing showed that LLIs are the best models of ageing with success, so this old population is the best population to be studied. On the average of onset of age-associated diseases, centenarians have been divided into three profiles, survivors, delayers, and escapers. Survivors had a diagnosis of an age-associated disease prior to the age of 80. Delayers were affected by an age-associated disease after the age of 80. Escapers attained 100^th^ year of life without the diagnosis of age-associated diseases [52]. Therefore, the extreme longevity is often characterized by a not unique and unequivocal phenotype, because there may be multiple routes to achieve exceptional longevity. However, each LLI can represent a model of “positive biology” by which it is possible to explain the biological mechanisms of health and well-being [44].

Changes associated with ageing affect the immune-inflammatory responses as shown by decline in immune function and increase in the systemic proinflammatory status, *i.e.*, immunosenescence, linked not only to the functional decline associated with the passage of time but also to antigen burden to which an individual has been exposed during lifetime [53]. The long-life chronic antigenic stress contributes to the chronic state of low-grade inflammation, inflammageing, observed in old people. Inflammageing is characterized by an increase in the levels of proinflammatory mediators. That, in turn, represents a negative prognostic factor for all causes of death. Oxidative stress plays an important role in determining and maintaining low-grade inflammation, which contributes to oxidative stress [54]. So, centenarians show an increase in many inflammatory molecules comparing to adults, but this condition is compensated by a concomitant activation of anti-inflammatory responses. This suggests that inflammageing may coexist with longevity especially if counterbalanced by an anti-inflammatory component. Someone who will (probably) become a centenarian should be able to keep inflammation down for longer [44].

The knowledge coming from these studies might provide valuable information to achieve healthy ageing by modulating the ageing rate and pointing out a sort of longevity signature. The identification of the factors that predispose to a successful ageing is of enormous interest for translational medicine [3].

In our paper, the results show that serum activity of MMP-2 increases in LLIs as compared to younger subjects. Furthermore, in LLIs, we find a positive correlation of MMP-2 with UA and an inverse correlation with CRP.

UA is the end product of endogenous and exogenous purine metabolism, and some epidemiological studies suggest that increased serum levels of UA are a risk factor for diseases where oxidative stress plays an important role. On the contrary, other evidence shows that UA might play a role as an antioxidant [46]. The possible explanation lies in the fact that UA might behave as oxidant getting older.

CRP, primarily secreted by the liver, is the most important biomarker of inflammation, commonly evaluated for monitoring treatment response and predicting long-term outcome in inflammatory diseases. The serum levels of CRP increase in an age-dependent manner and are good predictors of physical and cognitive performance and of the risk of mortality in both the entire old population and in successfully aged individuals [45].

Despite their name, MMP enzymes are not just bulk degraders of matrix proteins but they also provide a mechanism to add an additional layer of regulation to intercellular communication, including inflammation. In particular, MMP-2 regulates the processing of the chemokine monocyte-chemotactic protein 3 generating a chemokine receptor antagonist, which, in turn, might provide a regulation of the inflammatory signalling cascades directly exerted by MMP-2 [55]. More interestingly, MMP-2 activation is responsible for degrading the alarmin S100A9, thus limiting inflammation-inducing signals. It is relevant that alarmins are mediators of sterile inflammation in ageing and age-related diseases [56].

Therefore, the anti-inflammatory activity of MMP-2 can explain the negative and positive associations, respectively, with CRP and UA as well as the increased level in LLIs when compared to other younger groups. However, a note of caution in the interpretation of these results should be added because it has been demonstrated that antihypertensive drugs may reduce Pro-MMP-2 activity [57] and we do not have this information for most subjects under study.

MMP9 affects cholesterol metabolism, at least in part, through a MMP-9 plasma-secreted phospholipase A2 axis that affects the hepatic transcriptional responses to dietary cholesterol. Therefore, it has been proposed that the dysregulation of MMP-9 can contribute to the development of metabolic disorders that could, ultimately, lead to atherosclerosis and coronary heart disease [26]. The MMP-9 results show that their levels do not increase in LLIs and that the levels are highly variable in the whole population.

On the whole, the observed increase of MMP-2 in LLIs might suggest a positive role in the attainment of longevity. Interestingly, MMPs have been characterized as bifunctional proteins in Alzheimer's disease, with some of them, such as MMP-2 and MMP-9, displaying protective roles during disease progression, while others promote disease evolution [7].

However, it is difficult to make wide-ranging conclusions/assumptions based on these observations in view of the relatively small sample size of LLIs. Nonetheless, we believe that this is an important starting point for future larger-scale studies required to warrant these findings including the link with other systemic inflammatory and antioxidant markers.

## Figures and Tables

**Table 1:** Baseline characteristics of the studied cohort with haematochemical parameters.

		*n* = (%)	Group 1	Group 2	Group 3	Group 4	Group 5
Age			<40	40-64	65-89	90-94	≥95
*n* = (%)		154	29 (19%)	39 (25%)	50 (32%)	12 (8%)	24 (16%)

Gender	M	72 (47%)	13 (45%)	19 (49%)	28 (56%)	6 (50%)	6 (25%)
	F	82 (53%)	16 (55%)	20 (51%)	22 (44%)	6 (50%)	18 (75%)

CRP	<5 g/dL	127 (82%)	26 (90%)	34 (87%)	43 (86%)	6 (50%)	18 (75%)
	≥5 g/dL	27 (18%)	3 (10%)	5 (13%)	7 (14%)	6 (50%)	6 (25%)

UA	<2.4 g/dL	15 (10%)	4 (14%)	4 (10%)	1 (2%)	3 (25%)	3 (12%)
	2.4-7 g/dL	114 (74%)	23 (79%)	33 (85%)	37 (74%)	3 (25%)	18 (75%)
	>7 g/dL	25 (16%)	2 (7%)	2 (5%)	12 (24%)	6 (50%)	3 (13%)

CHO	<200 g/dL	113 (73%)	27 (93%)	22 (56%)	35 (70%)	10 (83%)	19 (79%)
	≥200 g/dL	41 (27%)	2 (7%)	17 (44%)	15 (30%)	2 (17%)	5 (21%)

LDL	<70 g/dL	15 (10%)	5 (17%)	0 (0%)	4 (8%)	2 (17%)	4 (17%)
	70-129 g/dL	106 (69%)	23 (79%)	23 (59%)	34 (68%)	8 (66%)	18 (75%)
	>70 g/dL	33 (21%)	1 (4%)	16 (41%)	12 (24%)	2 (17%)	2 (8%)

## Data Availability

The datasets generated and/or analysed during the current study are not publicly available due to privacy reasons but are available in anonymized form from the authors on reasonable request.
